# The Single-Bout Forearm Critical Force Test: A New Method to Establish Forearm Aerobic Metabolic Exercise Intensity and Capacity

**DOI:** 10.1371/journal.pone.0093481

**Published:** 2014-04-03

**Authors:** J. Mikhail Kellawan, Michael E. Tschakovsky

**Affiliations:** School of Kinesiology and Health Studies, Queen’s University, Kingston, Ontario, Canada; West Virginia University School of Medicine, United States of America

## Abstract

No non-invasive test exists for forearm exercise that allows identification of power-time relationship parameters (W′, critical power) and thereby identification of the heavy-severe exercise intensity boundary and scaling of aerobic metabolic exercise intensity. The aim of this study was to develop a maximal effort handgrip exercise test to estimate forearm critical force (fCF; force analog of power) and establish its repeatability and validity. Ten healthy males (20–43 years) completed two maximal effort rhythmic handgrip exercise tests (repeated maximal voluntary contractions (MVC); 1 s contraction-2 s relaxation for 600 s) on separate days. Exercise intensity was quantified via peak contraction force and contraction impulse. There was no systematic difference between test 1 and 2 for fCF_peak force_ (p = 0.11) or fCF_impulse_ (p = 0.76). Typical error was small for both fCF_peak force_ (15.3 N, 5.5%) and fCF_impulse_ (15.7 N⋅s, 6.8%), and test re-test correlations were strong (fCF_peak force_, r = 0.91, ICC = 0.94, p<0.01; fCF_impulse_, r = 0.92, ICC = 0.95, p<0.01). Seven of ten subjects also completed time-to-exhaustion tests (TTE) at target contraction force equal to 10%<fCF_peak force_ and 10%>fCF_peak force_. TTE predicted by W′ showed good agreement with actual TTE during the TTE tests (r = 0.97, ICC = 0.97, P<0.01; typical error 0.98 min, 12%; regression fit slope = 0.99 and y intercept not different from 0, p = 0.31). MVC did not predict fCF_peak force_ (p = 0.37), fCF_impulse_ (p = 0.49) or W′ (p = 0.15). In conclusion, the poor relationship between MVC and fCF or W′ illustrates the serious limitation of MVC in identifying metabolism-based exercise intensity zones. The maximal effort handgrip exercise test provides repeatable and valid estimates of fCF and should be used to normalize forearm aerobic metabolic exercise intensity instead of MVC.

## Introduction

The forearm handgrip exercise model is an important model for investigation of factors affecting, and mechanisms determining, O_2_ supply matching of exercising muscle O_2_ demand [1]–[3]. This is because such experiments are at best difficult, or at worst impossible, to perform in other exercise modalities. Furthermore, the forearm musculature has specific relevance for occupational settings and activities of daily living. In large muscle mass exercise models, non-invasive gas exchange can be used to identify ventilatory threshold and maximal oxygen uptake which can readily identify aerobic metabolic capacity, aerobic metabolic exercise intensity domains and the impact of interventions on these. Unfortunately there is currently no non-invasive exercise test that can provide this for the forearm handgrip exercise model.

Forearm exercise intensity is typically identified based on % of an individual’s maximum voluntary contraction (MVC) [2]–[4]. This is somewhat surprising since it has been well-established that %MVC is not related to metabolic exercise intensity domains [5], [6]. For example, Kent-Braun et al. [6] demonstrated that during progressive %MVC increases in dorsiflexion exercise the % MVC at which transitions across metabolic domains occur varied considerably between individuals. Saugen et al. [5] found marked between-subject differences in both time course and magnitude of PCr and pH changes during 40% MVC exercise in otherwise similar subjects.

A potential alternative approach would be to identify what has been traditionally referred to as the critical power (CP) which is the maximal power output that still results in a metabolic steady state characterized by a plateau in 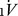
O_2_ and in inorganic phosphate [7]–[9]. In exercise above CP, exhaustion is precipitated by progressive fatigue and failure to stabilize metabolic state which may in part reflect depletion of a fixed anaerobic energy reserve, and likely also reflects the net effect of factors determining muscle force production for a given motor drive (i.e. factors determining muscle fatigue). The resulting fixed amount of work that can be performed above CP is termed W′. In exercise just below CP, exhaustion is precipitated by progressive fatigue despite a stable metabolic state. The stabilizing of PCr below but not above CP and the sensitivity of CP to manipulations of O_2_ delivery (increased with hyperoxia and decreased with hypoxia) indicate that CP is the maximal exercise intensity at which aerobic ATP production can completely match ATP demand [10]–[12]. These characteristics speak to the potential for CP as a means of quantifying muscle aerobic metabolic function, identifying aerobic exercise intensity domains, and assessing the impact of interventions on these in the forearm exercise model.

Recently, Burnley [13] validated a single bout, all-out intermittent isometric quadriceps contraction test to estimate CP quantified as knee extensor torque analog of CP. However, findings in this model cannot be assumed to apply to other small muscle mass exercise models. Accordingly, we have developed a maximal effort rhythmic isometric handgrip exercise test to estimate forearm critical intensity (fCF; force analog of CP). Such a test would allow both the identification of exercise intensity in terms of a measure of aerobic metabolic capacity, and the quantification of cross-sectional and both acute and chronic longitudinal intervention effects on said aerobic capacity. The aim of our study was to determine the repeatability and validity of the fCP estimated from this test. A secondary aim was to determine to what extent MVC was related to fCF and W′.

We hypothesized that fCF as quantified by the plateau in exercise intensity in the last 30 s of a 10 min maximal effort rhythmic isometric handgrip exercise test would demonstrate good between-day test-retest repeatability. Furthermore, we hypothesized that this exercise fCF plateau would be a valid representation of CP as reflected by a good agreement between time to exhaustion (TTE) during constant intensity exercise above fCF and TTE predicted for that exercise intensity based on the W′ estimated from the maximal effort test. Finally, we hypothesized that MVC would not be related to either fCF or W′.

## Materials and Methods

### Subjects

Ten healthy recreationally active males (20–43 years) volunteered to participate in the study.

### Ethics Statement

After receiving a complete verbal and written description of the experimental protocol and potential risks, each subject provided signed consent to the experimental procedures that were approved by the Queen’s University Health Sciences Research Ethics Board (HSREB) in accordance with the terms of the Declaration of Helsinki on research ethics.

### Experimental Design

All subjects experienced an initial familiarization visit. This was followed by two experimental visits separated by a minimum of 48 hours in which the subjects performed maximal effort rhythmic isometric handgrip exercise (i.e. fCF) tests. The first of these visits was within 24 hrs of the familiarization visit (visit details below). Seven subjects completed two additional visits separated by at least 24 hrs involving rhythmic handgrip exercise tests to exhaustion at a target intensity equal to 10% above fCF quantified as peak force (fCF_peak force_) or to a maximum of 20 min at 10% below fCF_peak force_ based on the higher of the two fCF_peak force_ estimates. All experimental sessions were conducted in a temperature-controlled laboratory (20–22°C) after a minimum of 2 hrs post-prandial and 12 hrs of abstaining from exercise and caffeine.

### Familiarization Trials

Familiarization trials were ∼30 min long. They involved having the participant perform maximal contractions at 1 s contraction to 2 s relaxation work cycle for three minutes at a time in order to become familiar with maintaining contraction intensity for 1 s based on visual feedback of continuously displayed force output (Powerlab, ADInstruments, Sydney, Australia) and audio and visual cues from a metronome.

### Maximal Effort Rhythmic Isometric Forearm Handgrip Critical Intensity Test (fCF)

**Figure 1:**
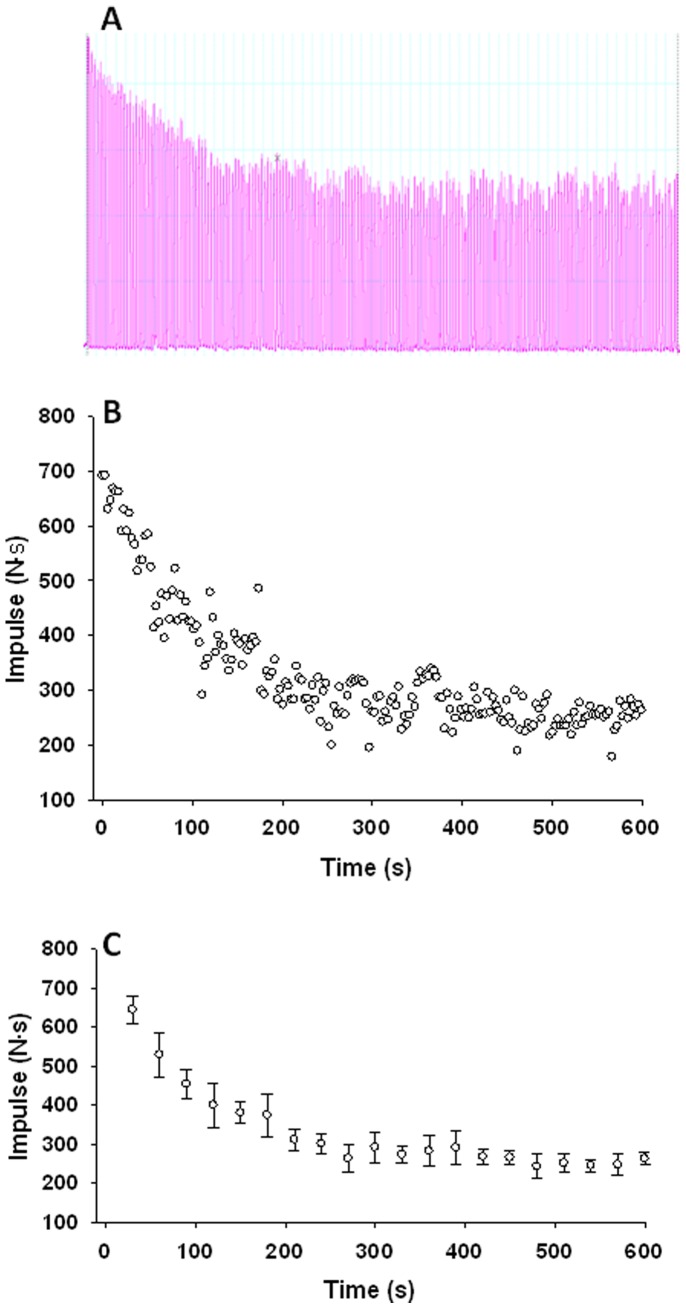
Force output during a 10 min maximal effort handgrip exercise test in a representative subject. **Panel A:** Raw force trace output **Panel B:** Impulse force of contraction plotted for all contractions during the test. **Panel C:** Impulse force averaged into 30 s time bins. Error bars indicate the contraction-to-contraction variability within each 30 s time bin.

Upon arrival at the laboratory, subjects lay supine with the experimental arm (left) extended 90^o^ at heart level as previously described [14]. After a period of acclimatization (∼5–10 min) subjects performed 3 maximal voluntary contraction (MVC) efforts separated by 1 minute. The highest of these was identified as the target contraction force for the maximal effort test. Data collection began with an initial 2 min period of quiet rest, followed by 10 min of rhythmic handgrip maximal voluntary contractions (1 s contraction to 2 s relaxation duty cycle). The 10 min duration of the maximal effort test was established during prior pilot work using a 10 min duration test in which it was observed that, while tests consistently resulted in subjects reaching a plateau by the last of the 10 min, some subjects did not reach a plateau prior to this time. Therefore 10 min was used for this duty cycle. It should be noted that the duration of the test would be expected to decrease with a higher contraction/relaxation duty cycle as the total work performed per unit time would be increased. Likewise it would be expected to increase with a lower contraction/relaxation duty cycle. Subjects observed their force output continuously displayed on a computer screen (Powerlab, ADInstruments, Sydney, Australia) (Fig. 1A) and attempted to reach their maximum force on every contraction. Subjects received constant verbal encouragement and coaching by a research assistant to achieve a “square wave” during each maximal voluntary contraction and to engage only the muscles of the forearm.

### Time-to-exhaustion Above and Below fCF_impulse_ Estimate

**Figure 2:**
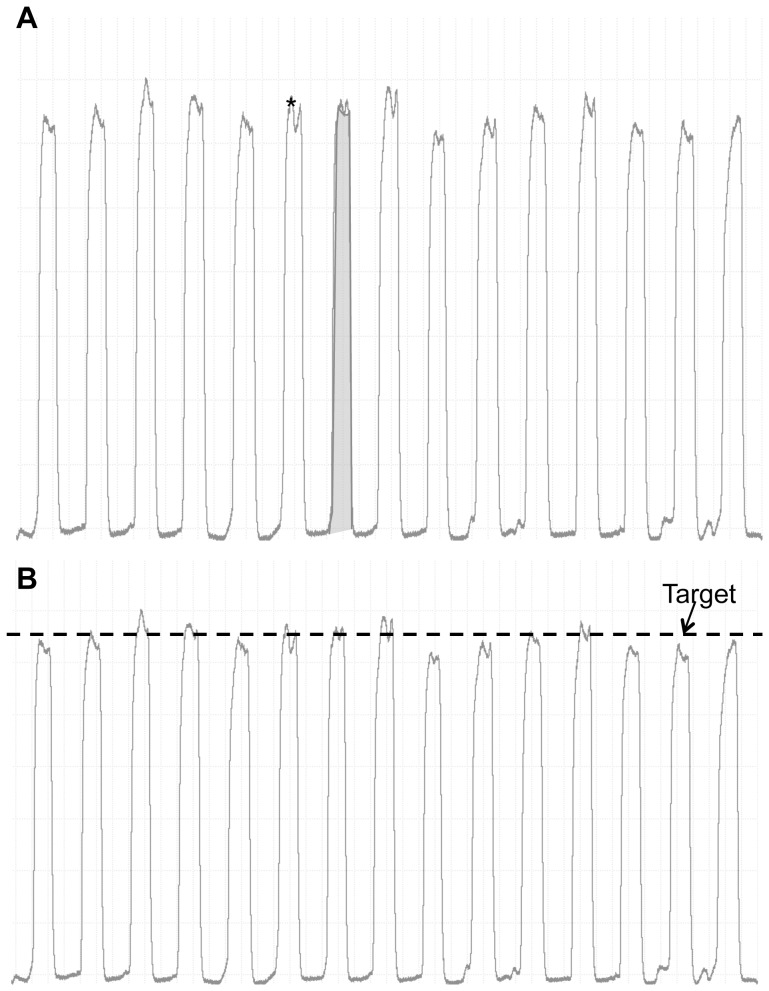
Force tracings from a representative subject. **Panel A:** Raw force trace depicting the data points used for calculation of the peak force (single *) and force impulse (shaded grey area) **Panel B:** Raw force trace during constant intensity handgrip exercise at target force of 10%>fCF_peak force_. Arrow indicates first of three consecutive contractions where target force was not achieved, and represents the time at which exhaustion occurred.

The constant intensity rhythmic handgrip exercise trials were performed at the same duty cycle as the maximal effort fCF test. The target force equal to 10% above or 10% below fCF_peak force_ was identified on the computer display screen with a target line. Time-to-exhaustion (TTE) was identified as the time of the first of three consecutive contraction efforts where the subject was unable to achieve the target force despite strong encouragement (Fig. 2B). All participants were stopped if they reached 20 min of exercise.

### Data Acquisition and Analysis

Handgrip force was obtained using an electronic handgrip dynamometer (ADInstruments, Sydney, Australia) connected to a data acquisition system sampling at 200 Hz (Powerlab, ADInstruments, Sydney, Australia) and recorded on a personal computer. For each contraction during a maximal effort test, we quantified both the peak contraction force and the integral of the force tracing (Impulse; see Fig. 2A).

**Figure 3:**
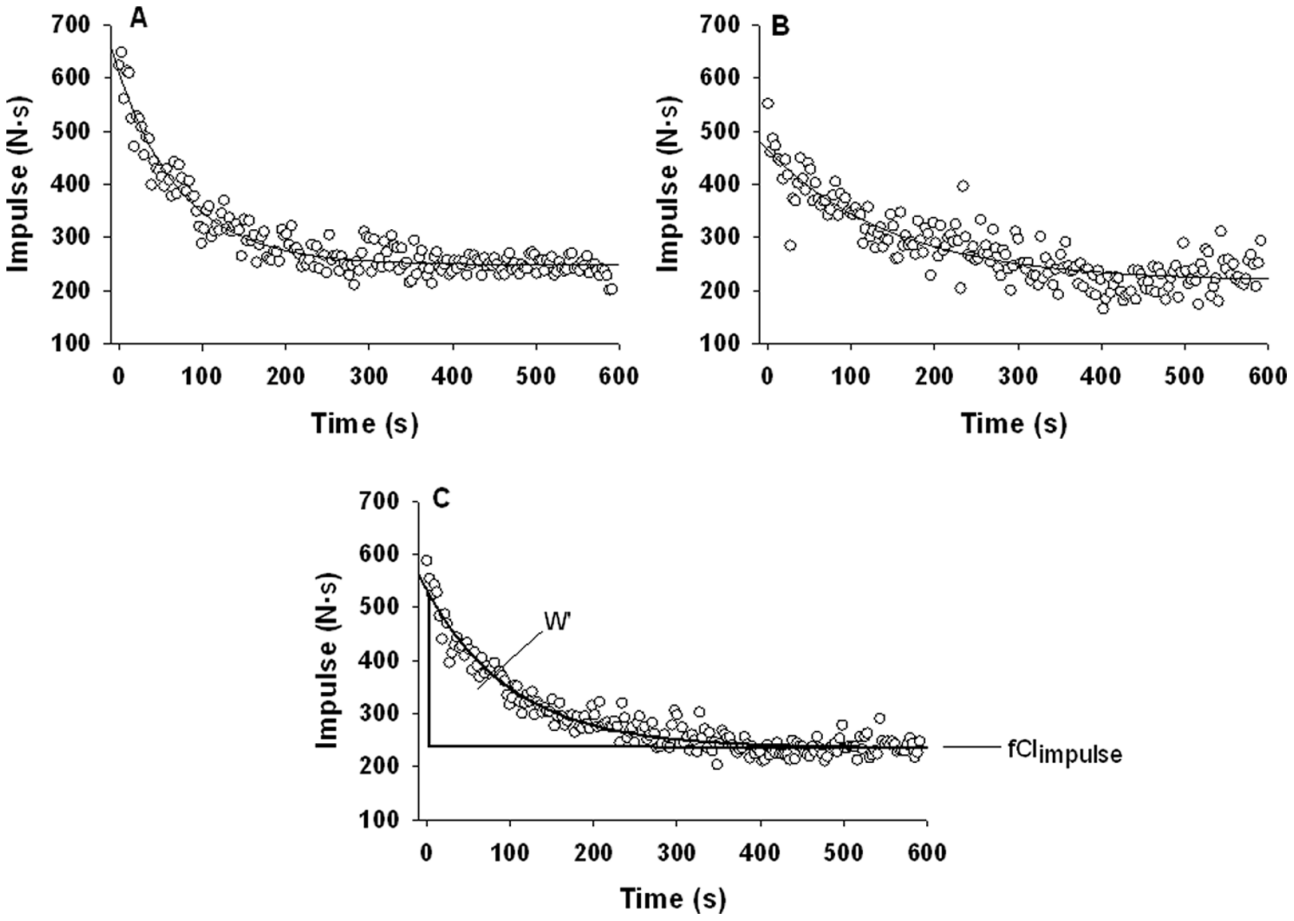
Curve fitting approach to determining fCF_impulse_ and W′ for a representative subject. **Panel A:** Test 1 plot of contraction impulses with line of best fit. **Panel B:** Test 2 plot of contraction impulses with line of best fit. **Panel C:** Average of Test 1 and Test 2, showing line of best fit, the fCF_impulse_, and the area between fCF_impulse_ and the line of best fit of the average contraction impulse which is W′.


To determine the fCF_peak_ _force_ and the fCF_impulse_ estimate for each test we obtained a line of best fit (LBF) for a 3 parameter exponential decay function (f = y0+a⋅exp^(−bx)^) fit to the plot of contraction impulse vs. time and contraction peak force vs. time (Sigmaplot 12.0 curve fit software; see Fig. 3 A). The last 30 s of the curve fit was used to quantify the fCF_impulse_ and the fCF_peak_ _force_. To quantify the W′ for each subject we averaged the two maximal effort tests to obtain a single plot of contraction impulse vs. time. We then fit this with the same 3 parameter exponential decay function and quantified fCF_impulse_ from the last 30 s of the curve fit**.** W′ was then determined by calculating the contraction impulse in excess of the fCF_impulse_ (EI_fCF Test_) for each contraction
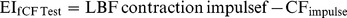
(1)and then calculating the sum of these (see Fig. 3C). To obtain a subject’s predicted TTE based on this W′, we first quantified the excess impulse for each contraction occurring during the constant supra-fCF_impulse_ intensity exercise test (EI_Constant Intensity Test_)
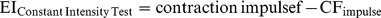
(2)and then calculated the average of these (AEIConstant Intensity Test). Since a contraction occurred every 3 seconds, a given contraction’s EIConstant Intensity Test would contribute to depletion of W′ every 3 seconds. Therefore we could calculate the predicted TTE (min)

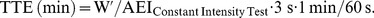
(3)

### Statistical Analysis

Repeatability of the fCF test was quantified as follows. First, a paired-t test was conducted to detect if there was a systematic difference between test 1 and test 2 for each of fCF_peak force_ and fCF_impulse_. Second, the standard error of measurement or typical error (the standard deviation (SD) of difference in scores/√2) expressed as absolute and as % of the grand mean (termed the coefficient of variation) was used in conjunction with the 95% limits of agreement (SD of the difference in scores multiplied by ± t_0.95, degrees of freedom_) to assess the magnitude by which test 1 and 2 typically differ [15], [16]. Finally, Pearson product and intra-class correlation coefficients (test 1 vs. test 2) were determined as a means of assessing how well the rank order of individuals was maintained between trials [16]. The same analyses were used to assess agreement between predicted and actual TTE. Simple linear regression was used to determine the strength of the relationship between MVC and each of fCF_impulse_, fCF_peak force_ and W′, where all parameters were plotted as the average of the two tests for each subject. Data are expressed as mean ± standard deviation (SD) unless otherwise indicated. Significance was set at p≤0.05.

## Results

### Force Profile of fCF Test

All subjects were able to complete the 10 min fCF test. The force decay to a plateau typical of all subjects is represented by the response of a subject in Fig. 1A and B. Within each trial, subjects reached the onset of a plateau somewhere between 420–540 s (Fig. 1C).

### Repeatability of the fCF Estimate

**Figure 4:**
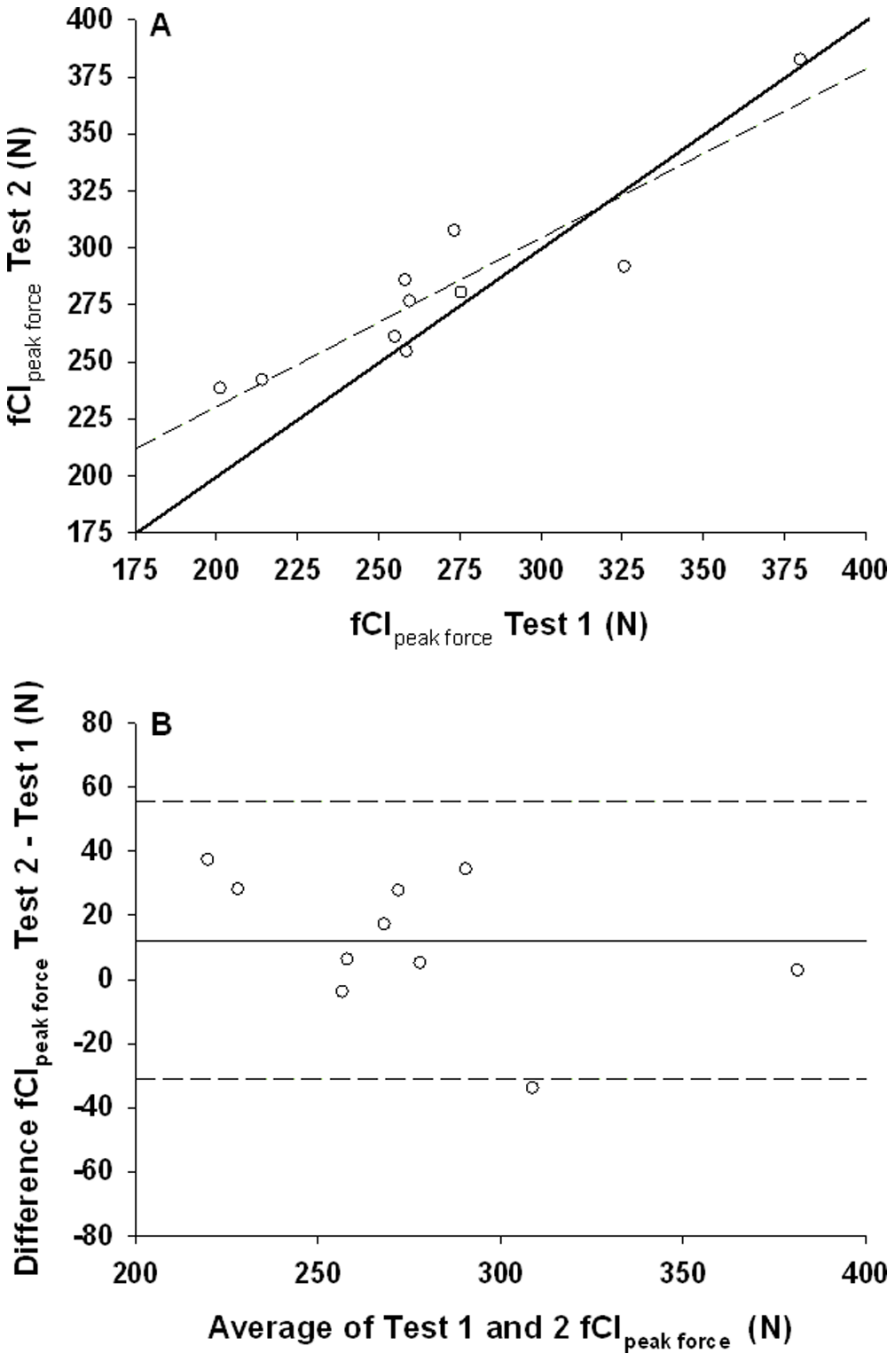
Test-retest correlations of fCF_peak force_. **Panel A:** fCF as peak force. Line of identity is shown as a solid line and the fitted regression as a dashed line. **Panel B:** Bland-Altman plot of fCF_peak force_. Mean difference between test 1 and test 2 (bias; solid line) ±43.2 N 95% limits of agreement (dashed lines).

**Figure 5:**
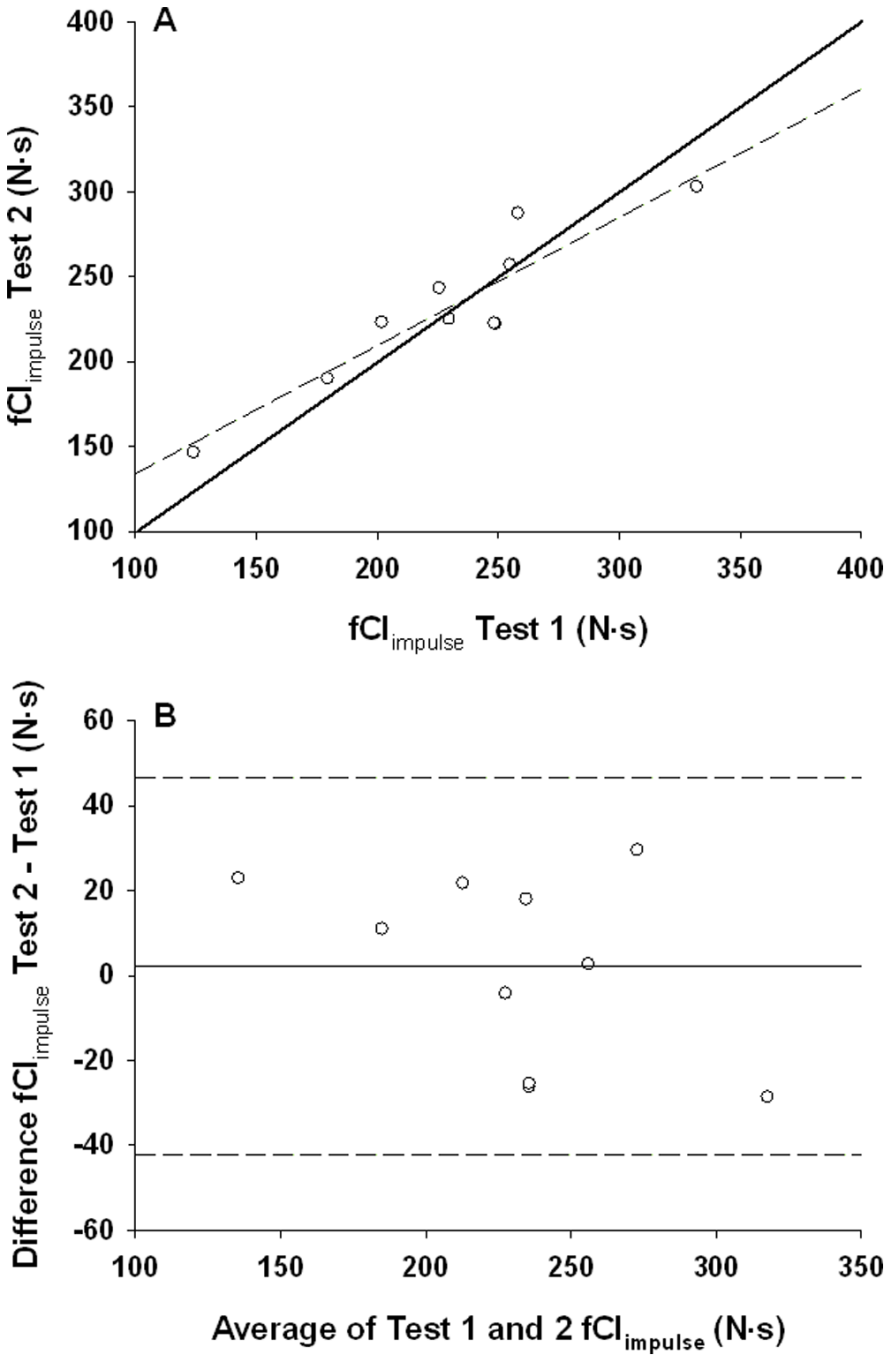
Test-retest correlations of fCF_impulse_. **Panel A:** fCF as force impulse. Line of identity is shown as a solid line and the fitted regression as a dashed line. **Panel B:** Bland-Altman plot of fCF_impulse_. Mean difference between test 1 and test 2 (bias; solid line). ±44.5 N⋅s 95% limits of agreement (dashed lines).

fCF_peak force_ and fCF_impulse_ repeatability is shown in Table 1 and 2 respectively. There was no systematic effect of trial on fCF_peak force_ (p = 0.11) or fCF_impulse_ (p = 0.76). The typical error expressed as absolute and % was small for fCF_peak force_ (15.3 N, 5.5%) and fCF_impulse_ (15.7 N⋅s, 6.8%). 95% limits of agreement were ±43.2 N with a bias of +12.3 for fCF_peak force_, and ±44.5 N⋅s with bias of +2.2 for fCF_impulse_ (Fig. 4B and 5B). Test-retest correlations using Pearson and intra-class correlations revealed strong positive relationships for both fCF_peak force_ (r = 0.91, ICC = 0.94, p<0.01) and fCF_impulse_, (r = 0.92, ICC = 0.95, p<0.01) (Fig. 4A and 5A).

**Table 1:** Forearm critical intensity peak force (fCF_peak force_) and its test-retest repeatability.

Subject	fCF_peak force_	fCF_peak force_	fCF_peak force_	SD (N)	Difference Score	CV (%)
	Test 1 (N)	Test 2 (N)	Mean (N)		Test 2–Test 1 (N)	
1	259.4	276.7	268.1	12.2	17.3	4.6
2	258.4	254.7	256.6	2.6	−3.7	1.0
3	201.1	238.5	219.8	26.5	37.5	12.0
4	213.9	242.2	228.1	20.0	28.2	8.7
5	379.8	382.8	381.3	2.1	2.9	0.5
6	275.3	280.6	277.9	3.7	5.3	1.3
7	254.8	261.1	258.0	4.5	6.3	1.7
8	273.2	307.7	290.4	24.4	34.5	8.4
9	258.0	285.9	271.9	19.7	27.9	7.2
10	325.6	291.9	308.7	23.8	−33.7	7.7
Mean	270.0	282.2	276.1	14.0	12.2	5.5
(SD)	(51.3)	(41.7)	(45.5)	(10)	(21.6)	–

CV – coefficient of variation. The “mean” CV is the typical error as a % of the grand mean.

**Table 2:** Forearm critical intensity impulse (fCF_impulse_) and its test-retest repeatability.

Subject	fCF_impulse_	fCF_impulse_	fCF_impulse_	SD (N)	Difference Score	CV (%)
	Test 1 (N)	Test 2 (N)	Mean (N)		Test 2–Test 1 (N)	
1	201.7	223.5	212.6	15.4	21.8	7.2
2	248.6	222.4	235.5	18.5	−26.2	7.9
3	179.3	190.3	184.8	7.8	11.1	4.2
4	123.8	146.8	135.3	16.3	23.0	12.0
5	258.0	287.6	272.8	20.9	29.6	7.7
6	254.6	257.4	256.0	1.9	2.7	0.8
7	229.5	225.3	227.4	2.9	−4.1	1.3
8	225.4	243.5	234.5	12.8	18.1	5.5
9	248.2	222.9	235.5	17.9	−25.3	7.6
10	331.9	303.3	317.6	20.2	−28.6	6.4
Mean	230.1	232.3	231.2	13.5	2.2	6.8
(SD)	(54.9)	(45.0)	(48.9)	(7.0)	(22.2)	–

CV – coefficient of variation. The “mean” CV is the typical error as a % of the grand mean.

### Constant Intensity Tests

For the constant intensity test 6 of 7 subjects were able to complete 20 min of rhythmic forearm exercise when the target force was 10% below fCF_peak force_, whereas only 1 of 7 could complete 20 min exercise when the target force was 10% above fCF_peak force_ (TTE 18.0±5.0 min vs. 10.3±5.9, p<0.01). Table 3 presents W′ and the contraction impulse data from the constant intensity tests where target force was 10% above fCF_peak force_ as well as the data for subject 5 where the target was 10% below fCF_peak force_ but his average contraction impulse during this constant intensity test actually exceeded the fCF_impulse_, and therefore resulted in TTE well below 20 min. The actual constant exercise intensity contraction impulses performed for tests used in obtaining predicted TTE exceeded fCF_impulse_ by 59.9±36.0 N⋅s (27.7±18.8%).

**Table 3:** Individual W′ estimated from maximal effort test, and average contraction impulse above fCF_impulse_ in the constant intensity exercise tests where subjects exercised above fCF_impulse_.

Subject # -	W′	fCF_impulse_	Impulse in Excessof fCF_impulse_ (N+s)	Impulse in Excessof fCF_impulse_ (%)
Constant Intensity Test	(N+s)	(N+s)		
1 – Above fCF_impulse_	6277	212.9	28.1	13.2
2 – Above fCF_impulse_	10053	236.9	99.0	42.8
3 – Above fCF_impulse_	5477	186.4	28.4	15.2
4 – Above fCF_impulse_	10551	138.6	86.7	62.6
5 – Below fCF_impulse_	6133	276.0	114.9	41.6
5 – Above fCF_impulse_	6133	276.0	57.3	20.8
6 – Above fCF_impulse_	11815	256.6	44.4	17.3
7 – Above fCF_impulse_	6075	231.2	20.7	9.0
Mean	7814	217.8	59.9	27.7
(SD)	(2536)	(46.2)	(36.0)	(18.8)

Above and Below fCF_impulse_ – data from constant load test where target force was 10% above or 10% below the highest fCF_peak force_ of the two maximal effort tests. Subject 5 exercised above his fCF_impulse_ during the constant intensity 10% below test and therefore these data have been included.

### Time to Exhaustion (TTE): Predicted vs. Actual

**Figure 6:**
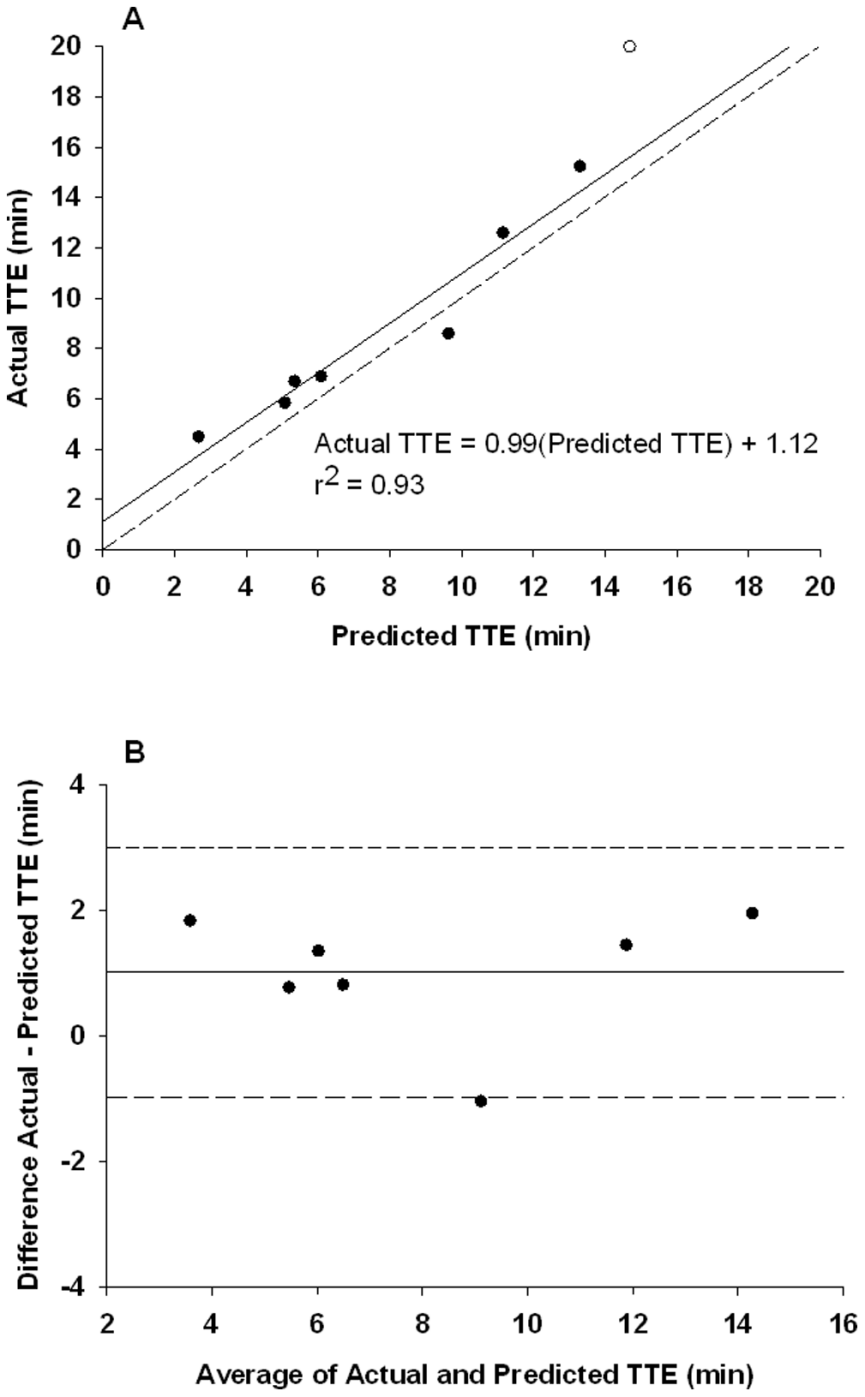
Predicted vs. actual time to exhaustion (TTE) in constant supra-fCF_impulse_ intensity exercise tests. **Panel A:** Regression of predicted vs. actual TTE (closed circles), excluding data for subject 7 whose actual TTE was constrained by the 20 min limit of test duration (open circle). **Panel B:** Bland-Altman plot of TTE. Mean difference between predicted and actual TTE (solid line). ±1.97 min 95% limits of agreement (dashed lines).

Agreement between predicted TTE calculated as per equation [3], and actual TTE during constant intensity exercise is shown in Table 4 and Figure 6. Subject 5 exercised above his fCF_impulse_ during the 10% below fCF_peak force_ target constant intensity test and therefore their TTE data from this test is included. Subject 7 reached the 20 minute end test point during their 10% above fCF_peak force_ target constant intensity test at which point they were stopped. They are therefore not included in the analysis of predicted vs actual TTE agreement. There was no difference at the group level between predicted and actual TTE (p = 0.25). The typical error was 0.98 min (12%) and the limits of agreement were; bias 1.38±1.87 min. Pearson and intra-class correlations were strong (r = 0.97, ICC = 0.97, P<0.01). For the regression fit, the slope was 0.99 and the y-intercept was not significantly different from 0 (1.12 min, p = 0.31).

**Table 4:** Predicted and actual time to exhaustion (TTE) for constant intensity exercise tests where subjects ended up exercising above fCF_impulse_.

Subject # - Constant	TTE_predicted_	TTE_actual_	Mean of TTE_predicted and actual_	SD	Difference Score	CV
Intensity Test	(min)	(min)		(min)	TTE_actual_ - TTE_predicted_	(%)
1 – Above fCF_impulse_	11.2	12.6	11.9	1.0	1.4	8.3
2 – Above fCF_impulse_	5.1	5.9	5.5	0.5	0.8	10.0
3 – Above fCF_impulse_	9.6	8.6	9.1	0.7	−1.0	8.1
4 – Above fCF_impulse_	6.1	6.9	6.5	0.6	0.8	8.7
5 – Above fCF_impulse_	4.5	2.7	3.6	1.3	−1.8	35.4
5 – Below fCF_impulse_	5.4	6.7	6.0	1.0	1.4	15.8
6 – Above fCF_impulse_	13.3	15.3	14.3	1.4	2.0	9.7
7 – Above fCF_impulse_	*14.7*	*20+*	*17.4*	*3.7+*	*5.3+*	*21.6+*
Mean	7.9	8.4	8.1	0.9	0.5	12.1
(SD)	(3.5)	(4.3)	(3.8)	(0.3)	(1.4)	–

CV – coefficient of variation. The “mean” CV is the typical error as a % of the grand mean. Constant Intensity Test – Above is the test where the target contraction force was 10% >fCF_peak force_. Constant Intensity Test – Below is the test where the target contraction force was 10% <fCF_peak force_. Subject 5 exercised above fCF_impulse_ during the constant intensity test where the target was below fCF_peak force_. Subject 7 data in italics is not included in the Mean±SD.

### Relationship of MVC to fCF, W′ and Incremental Exercise Peak Intensity

**Figure 7:**
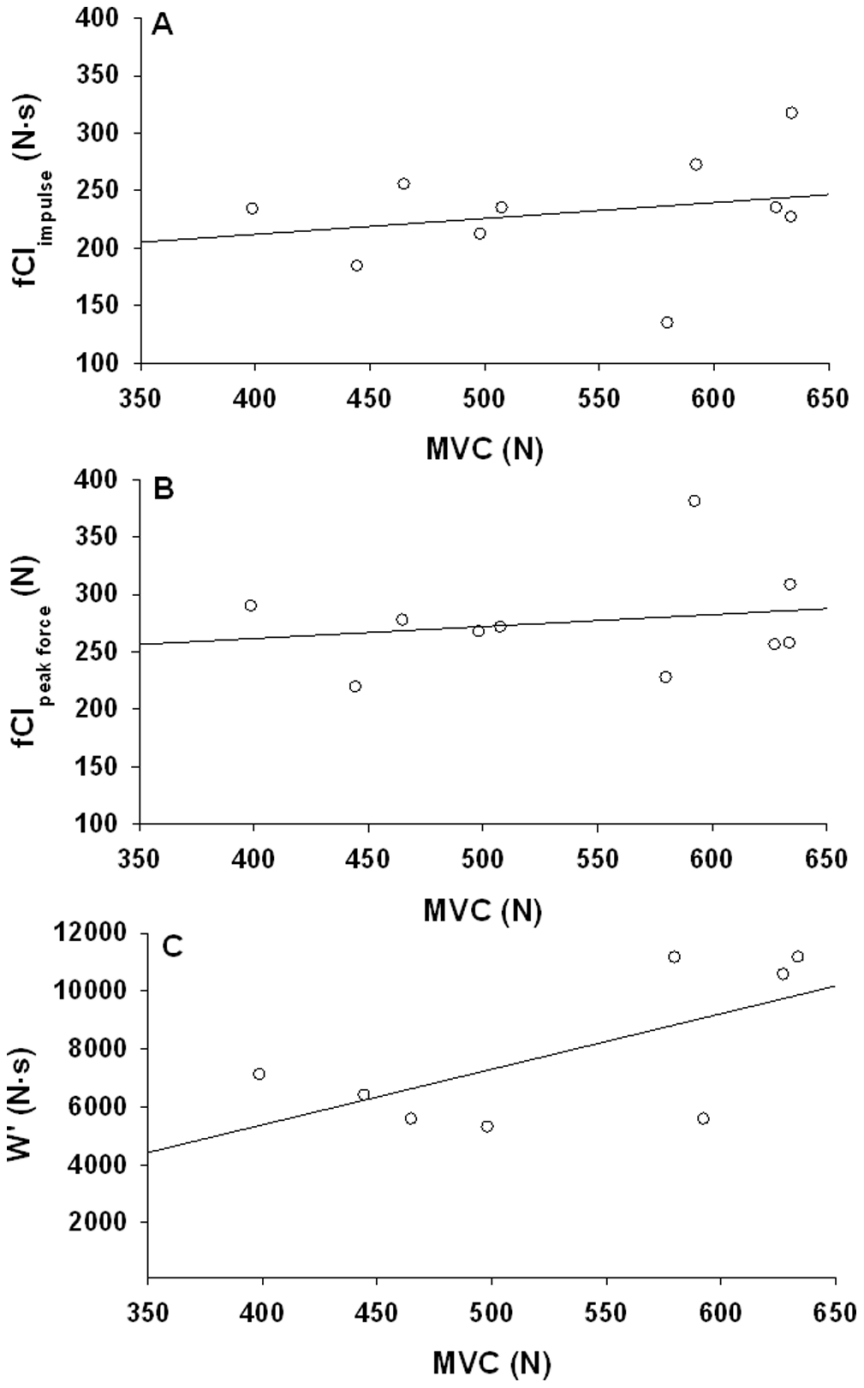
Relationship between Maximum Voluntary Contraction (MVC) and Panel A: fCF_impulse_. r^2^ = 0.06, p = 0.490, **Panel B:** fCF_peak force_ r^2^ = 0.10, P = 0.37 and **Panel C:** W′ r^2^ = 0.37, p = 0.15.

There was no statistically significant relationship between MVC and fCF_impulse_ (r^2^ = 0.06, p = 0.490), fCF_peak force_ (r^2^ = 0.10, P = 0.37) or W′ (r^2^ = 0.37, p = 0.15) (see Fig. 7). Five subjects in this study also performed incremental ramp test to failure as part of another study, and again MVC was not related to their incremental ramp test peak exercise intensity (r^2^ = 0.006, p = 0.9).

## Discussion

The novel findings of this study were: 1) forearm maximal effort exercise resulted in the same type of force decay to a stable plateau as previously demonstrated for single-leg all out exercise [13], 2) the stable force plateau had good repeatability both as fCF_peak force_ and fCF_impulse_, 3) predicted TTE for the constant supra-fCF_impulse_ intensity exercise test based on the fCF test W′ showed good agreement with actual TTE, 4) MVC showed no association with fCF. Taken together, these findings support the reliability and validity of the single bout maximal effort handgrip test in identifying fCF as well as W′ and argue for its use instead of MVC for identification of exercise intensity when considerations of aerobic metabolism are important in studies using the forearm exercise model.

### Characteristics of fCF Test Force

The force decay profile for the single bout maximal effort handgrip exercise test was consistent with previous maximal effort tests used to estimate critical torque or CP [11], [13]. The longer time to plateau in our study is expected, since duty cycle was less than in cycling or single knee extension [11], [13] and less frequent contractions would result in more time required to deplete W′.

### Repeatability of Single Bout fCF Test

Our repeatability analysis demonstrated small within-subject test-retest variation, small change in the test-retest group mean, and a high test-retest correlation, all indicators of good repeatability [17], [18]. To allow direct comparison between our study and others that used different parameters and demonstrating widely varying magnitude of response [17] we expressed our indices of repeatability in percent. Our findings were similar to previous findings during maximal effort cycling (CV 3%, ICC 0.99) and a meta-analysis of CP (maximum aerobic power, running or cycling exercise) estimates derived from time-to-exhaustion tests (CV, −0.5–7.6%, Δ mean, −2.2–5.8%) [17], [19]. However, CP estimates from traditional multiple bout time-to-exhaustion tests have not always been found to be reliable [20]. Large CV’s (>15%) for time-to-exhaustion tests at a given exercise intensity have been previously reported [21], [22]. If each data point of a multiple time-to-exhaustion test has considerable variability, then a curve fit based on those points would be susceptible to increased variability. The single bout maximal effort test eliminates this problem.

### Validity of the Single Bout fCF Test

Traditionally, curve fitting of data from three to five fixed power output time-to-exhaustion tests in order to identify the asymptote of the power-duration relationship has been used to identify CP. Therefore, the approach to validating single maximal effort test identification of CP has been to compare results between the two tests in the same individuals [11], [13].

As identified by Barker et al. [23], CP is a metabolic rate. Furthermore, it is a metabolic rate that can be sustained because of stabilization of PCr, 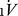
O_2_, and pH as identified by Jones et al. [8]. As cleverly proposed by Burnley et al. [19], the basis for a single maximal effort test to result in power output plateauing at CP is the relationship between W′, power output of the task, and CP:
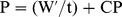
(4)such that during continued maximal effort exercise, W′ would “reduce to zero, at which point the highest possible power output would be equal to CP” [19]. Consistent with this, work from this group went on to confirm that a single “all-out” exercise test in both knee extension [13] and cycle ergometer [11] exercise modalities yielded CP estimations that were in excellent agreement with those identified from the traditional multiple fixed power output time-to-exhaustion trial approach. As summarized by Jones et al. [24], the amount of work that can be performed above CP is “not dependent on the chosen work rate above CP”. This means that whether W′ is depleted over the course of an all-out exercise bout, or a constant intensity exercise bout above CP, it will be quantitatively the same. This is the basis for being able to utilize W′ to predict time to failure during exercise at a constant intensity above CP.

Based on this we reasoned that, if the contraction impulse plateau in our single bout maximal effort forearm exercise test was a valid estimate of fCF_impulse_, then an estimation of W′ from that test would necessarily allow prediction of time-to-exhaustion (TTE) during exercise at a constant intensity above the impulse plateau. It is necessary to use the contraction impulse rather than contraction peak force, as exercise intensity has both a force and a time domain. Therefore, to assess validity, we calculated a predicted TTE for the constant intensity test that was based on a W′ calculated from the excess force impulse during the maximal effort fCF testing and compared it with actual TTE in the constant intensity test.

For this TTE analysis we included all constant intensity exercise tests where exhaustion occurred before 20 minutes. This was the case for subject 5′s constant intensity test where the target contraction force was 10% below fCF_peak force_. The subject actually performed exercise at an intensity that was above his fCF_impulse._ This resulted in task failure before 20 minutes as a consequence of W′ depletion. Likewise, we excluded data from subject 7′s constant intensity exercise test where the target contraction force was 10% above fCF_peak force_, because they reached the 20 minute mark where exercise was stopped for all subjects (see Fig. 6) and therefore their data point does not represent the actual TTE during their test. This was considerably greater than his predicted time to exhaustion of 14.7 minutes. It is not clear why in this one instance there was such disagreement between predicted and actual TTE, since the average recorded contraction force impulse during this test was greater (see Table 3 Subject 7) than the fCF_impulse_ determined from the maximal effort test. There are (at least) three potential interpretations of the TTE data. First, for one subject out of 8, the single bout maximal effort forearm exercise test we developed is unable to provide an estimate of W′, and therefore the fCF_impulse_, despite excellent repeatability (CV 1.3%; see Table 1 Subject 7), is not valid. Second, the test we developed does not provide valid estimation of fCF_impulse_ and by chance we had 7 tests out of 8 where agreement between actual and predicted TTE was good. Third, it is possible that the position of the handgripper in the subject’s hands was different in the constant supra-fCF_impulse_ test vs. the two single bout maximal effort tests from which fCF_impulse_ was determined. A difference in position of the handgripper can alter the mechanical advantage such that the actual muscle contraction force to achieve a given handgrip force can be different. In the case of subject 7, if mechanical advantage was improved during the constant supra-fCF_impulse_ exercise test, the actual muscle contraction force was less than that quantified from the recorded force tracing. Given that the recorded force tracing indicated this subject was exercising only 9% above fCF_impulse_ it would not require much of a reduction in actual vs. recorded force for the subject to be at or below their fCF_impulse_ during this test. Given that in all other cases the predicted vs. actual TTE was close to the line of identity, this third possibility would seem to be the most likely explanation.

Our predicted vs actual TTE findings are in good agreement with those of Jones et al. [8] who used the traditional multiple bout time to exhaustion protocol for identifying the critical power and quantifying the W′ (curvature constant of the hyperbolic relationship between fixed power output vs. time using 3 or 4 TTE tests for each subject). Subjects then performed constant intensity exercise tests on separate days at 10% above and 10% below the critical power estimate. All subjects reached 20 min of exercise at 10% below critical power at which time they were stopped. For the 10% above critical power tests the TTE ranged from 6.9–23.8 min such that predicted TTE was similar to actual TTE (mean of 15.1±3.3 min vs. 14±7.1 min; r^2^ = 0.76).

We also observed no difference between actual TTE 8.4+/−4.3 vs. predicted TTE 7.9+/−3.5 min, with a strong correlation between predicted and actual TTE of r^2^ = 0.93. The fact that our single bout maximal effort forearm critical intensity test provided estimates of W′ and fCF_impulse_ that allowed as good a prediction of TTE as the traditional multiple bout TTE derived critical power and W′ estimation further supports the validity of this test.

### % MVC vs. fCF as a Measure of Relative Aerobic Metabolic Exercise Intensity

We observed a poor relationship between MVC and fCF. This is consistent with MVC force being a function of the cross-sectional area of muscle fibres involved, the percentage of fast-twitch muscle fibres involved, and the number of motor units recruited/activated, not metabolic demand or oxygen delivery [25]–[27]. By extension, a given %MVC would not represent exercise intensity relative to aerobic metabolic capacity and therefore could not provide a valid identification of aerobic metabolic exercise intensity domains.

This has been previously confirmed by the work of Saugen et al. [5] and Kent-Braun et al. [6]. Their findings reinforce that myocellular environments and aerobic metabolism can be drastically different between individuals during exercise at the same % MVC. Therefore, selection of exercise intensities relative to an individual’s MVC could lead to subjects exercising in different aerobic metabolic exercise intensity domains, confounding interpretation of results. In contrast, CP (in the current study fCF_impulse_) represents an exercise intensity that reflects functional aerobic metabolic capacity and delineates steady state from non-steady state exercise [24], [28]. Our findings argue for the abandonment of MVC for establishing exercise intensities in the forearm exercise model, and its replacement by the newly developed fCF test.

### Potential Limitations

This study utilized a 1 s contraction: 2 s relaxation rhythmic isometric contraction exercise protocol. That the repeatability we found would also occur if exercise was performed with other duty cycles (eg. 1∶1, 2∶1 etc…) or with other contraction durations without a change in duty cycle (eg. 2∶4, 3∶6) cannot be claimed with certainty. However, given that critical intensity of a single all-out test shows excellent agreement with multiple bout time to exhaustion tests in isokinetic knee extension exercise with a 3∶2 duty cycle [13], and in cycle ergometer exercise [11], and that critical intensity is a metabolic rate that is consistent between cycle cadences with differing 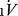
O_2_ cost of power output [23], it would not be expected that contraction protocol would affect repeatability. Likewise for other duty cycles, or contraction durations without change in duty cycle, to result in a single bout maximal effort that does not provide a valid estimate of fCF would require that the nature of the contraction protocol disrupts the physiological underpinning of W′, fCF_impulse_, and of their relationship.

Another important consideration is that of the effect of isometric contraction duration on the 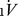
O_2_ cost under the same tension-time integral (TTI; representing impulse per unit time). It has been well established in animal models that 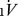
O_2_ is higher during shorter vs. longer duration rhythmic isometric contractions [29], [30] under the same TTI. This is believed to be a function of the increased ATP cost of ion transport with more frequent muscle activation and relaxation events [29]. It has also been shown in a cycle ergometer exercise model using different cycle cadences, where faster cadence results in an increased 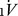
O_2_ cost at a given external power output, that power output but not 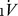
O_2_ at CP differs [23], [31]. Taken together, these findings raise the possibility that comparison of fCF_impulse_ between contraction protocols that differ in contraction duration may be problematic.

In contrast, if one were to compare fCF_impulse_ between tests where duty cycles differ but contraction duration is the same, then differences in 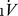
O_2_ cost of a given fCF_impulse_ would not be expected to differ and comparison may be possible. One would simply expect that, when relaxation duration is increased for the same contraction duration, the peak force of the contraction at fCF_impulse_ would be increased.

Finally, it is important to realize that when conducting voluntary isometric handgrip exercise tests that the ability of a subject to perfectly execute a 1 second contraction as a square wave of force production to target force is not possible. Thus, there is the potential for variability between subjects in the actual contraction force amplitude and duration such that the peak force and force impulse varies from the target. Unfortunately, this cannot be determined until offline analysis of the contraction force impulse after completion of the exercise test. The exercise task performance of subject 5 in the 10% above fCF_impulse_ is an example of when this variability can have implications for data interpretation. Familiarization of the subject with the exercise task and ongoing monitoring and correction during the actual exercise can reduce this issue to some degree. Nevertheless, quantification of the peak and impulse performed in an exercise test is essential to account for this potential confound when interpreting findings.

### Conclusions

In conclusion, we have established that a single bout 10 min maximal effort handgrip exercise test demonstrates good repeatability of fCF_peak force_ and fCF_impulse_ estimated from the last 30 s (or 10 contractions) of the test. The good agreement of predicted and actual TTE supports the validity of the fCF_impulse_ estimate and its usefulness in quantifying W′. The poor relationship of MVC with fCF is consistent with MVC being a poor means for identifying relative aerobic metabolic exercise intensity between subjects. Therefore, the fCF rather than MVC should be utilized in the human forearm exercise model to identify exercise intensity domains, and characterize aerobic metabolic demand when aerobic metabolic considerations are important.
